# Impact of early versus late *a*rtificial *r*upture of *m*embranes during oxytocin induction of labour on the incidence of chorioamnionitis: a randomised controlled *trial* (ARM trial)

**DOI:** 10.1186/s13063-025-08722-z

**Published:** 2025-01-22

**Authors:** Meghan G. Hill, Michelle R. Wise, Emmanuelle Pauleau, Beatrice Treadwell, Lynn Sadler

**Affiliations:** 1 Department of Obstetrics and Gynaecology, The University of Auckland, Level 1, Building 507, Grafton, Auckland, 1023 New Zealand; 2 Women’s Health, Te Whatu Ora Te Toka Tumai Auckland, 2 Park Road, Grafton, Auckland, 1023 New Zealand

**Keywords:** Artificial rupture of membranes, Amniotomy, Chorioamnionitis, Oxytocin, Labour induction

## Abstract

**Background:**

The approach to induction of labour differs internationally, with timing of amniotomy being controversial. Some institutions favour performing artificial rupture of membranes prior to commencement of oxytocin infusion, with the belief that the labour will progress more efficiently. In other institutions, the approach recommended is for oxytocin infusion with intact amniotic membranes until the person has reached the active phase of labour, citing risk of infection with early amniotomy. Current evidence is inconclusive. We are performing a randomised controlled trial assessing whether delaying amniotomy until the active phase of labour can decrease the rate of chorioamnionitis.

**Methods:**

This is a randomised controlled trial at a single centre in New Zealand. Pregnant people undergoing induction of labour at ≥ 37 weeks gestational age with intact membranes and a singleton gestation are eligible for the trial. Participants are randomised to ‘Early’ amniotomy, at the commencement of oxytocin infusion, or to ‘Late’ amniotomy, when they have reached a cervical dilation of 6 or more centimetres or when they have been receiving oxytocin infusion for 12 h. The primary outcome of the trial is chorioamnionitis. To detect a decrease in chorioamnionitis from 9 to 3% with a power of 80% and a 95% CI, we will require 488 participants in total, randomised in a 1:1 ratio.

**Discussion:**

If delaying amniotomy reduces the rate of chorioamnionitis, this is important to inform future practice. Chorioamnionitis entails risk to both the pregnant person and the fetus and is an important contributor to neonatal sepsis, neonatal intensive care unit admission, maternal sepsis, caesarean, wound infection and postoperative infective complications. Conversely, if the rate of chorioamnionitis is not affected by timing of amniotomy, this will allow for safe individualization of care.

**Trial registration:**

The trial is registered on the Australian and New Zealand Clinical Trials Registry, anzctr.org.au. Full registry title is ‘Impact of early versus late artificial rupture of membranes during oxytocin induction of labour on the incidence of chorioamnionitis: A randomised controlled trial’. Trial ID: ACTRN12621000405819. Date registered 14 April 2021.

## Administrative information

**Table:** 

Title {1}	Impact of early versus late **A**rtificial **R**upture of **M**embranes during oxytocin induction of labour on the incidence of chorioamnionitis: A randomised controlled **trial (ARM Trial)**
Trial Registration {2a and 2b}	Australian and New Zealand Clinical Trials Registry (ANZCTR) #ACTRN12621000405819 All items from the WHO Trial Registration Data Set can be located within this protocol
Protocol Version {3}	2
Funding {4}	University of Auckland, FDRF, Financial University of Auckland, Nurture, Financial University of Auckland, Nurture, Financial Lottery Health Research Grants, Financial
Author Details {5a}	Meghan G. Hill, MBBS, MS Department of Obstetrics and Gynaecology The University of Auckland Level 1, Building 507 Grafton, Auckland, New Zealand, 1023 Meghan.hill@auckland.ac.nz Michelle R Wise Department of Obstetrics and Gynaecology The University of Auckland Level 1, Building 507 Grafton, Auckland, New Zealand, 1023 m.wise@auckland.ac.nz Emmanuelle Pauleau Women’s Health Te Whatu Ora Te Toka Tumai Auckland 2 Park Road Grafton, Auckland, New Zealand, 1023 EPauleau@adhb.govt.nz Beatrice Treadwell Women’s Health Te Whatu Ora Te Toka Tumai Auckland 2 Park Road Grafton, Auckland, New Zealand, 1023 BeatriceT@adhb.govt.nz Lynn Sadler Women’s Health Te Whatu Ora Te Toka Tumai Auckland 2 Park Road Grafton, Auckland, New Zealand, 1023 lynns@adhb.govt.nz
Name and Contact Information for the trial sponsor {5b}	Te Tari Rautaki Rangahau Matatika / Office of Research Strategy and Integrity (ORSI) Waipapa Taumata Rau / University of Auckland Phone: + 64 9 373 7599, Extension 85,405
Role of sponsor {5c}	The University of Auckland is the study sponsor. The University is a public institution and indemnifies the trial. The sponsor did not have a role in study design, collection, management and analysis of data, interpretation of data, writing of the report or decisions regarding publication

## Introduction

### Background and rationale {6a}

Induction of labour (IOL) is one of the most common procedures performed in pregnant people, with approximately 40% of labours being induced at Te Toka Tumai Auckland [1]. There are multiple induction agents that may be used for IOL, including prostaglandins, catheters and oxytocin infusion [2–5]. There appears to be regional variation in induction and labour management in relation to artificial rupture of membranes (ARM), with some centres performing ARM liberally, while other centres are quite restrictive in the practice. People undergoing induction of labour in New Zealand commonly have an ARM early during the process, often immediately prior to oxytocin infusion. This differs from other settings where ARM is performed after the patient enters active labour, or not at all (meaning that spontaneous rupture of membranes [SRM] is awaited). Two Cochrane reviews have investigated the role of ARM. One review assessed performance of ARM during spontaneous labour and one assessed ARM to induce labour [6, 7]. Neither identified a clear benefit to ARM and hence it was not recommended by either group of authors [6, 7].

One potential issue with performing an early ARM is that the protective barrier between the uterine cavity and the vagina (the amniotic membrane) is now interrupted, allowing for ascent of bacteria from the vagina into the uterus. Moreover, this practice permits the fluid ‘cushion’ surrounding the fetus to be released and theoretically provides a greater chance for umbilical cord compression, leading to fetal heart rate decelerations. These theories that lead to a concern about early amniotomy are supported by evidence from three small trials [8–10].

The first trial, involving 209 participants in the United States, identified a marked difference in people undergoing amniotomy in rates of chorioamnionitis (22.6% versus 6.8%, *p* = 0.002 in the early ARM versus late ARM groups, respectively) and variable decelerations (19.6% versus 6.4%, *p* = 0.08 in the early ARM versus later ARM groups, respectively) [8]. The second trial including 168 people recruited in Israel identified an elevated risk of intrapartum fever (which we consider to be a surrogate for chorioamnionitis) in the early ARM group (8.7% versus 2.3%, RR 1.69; 95% CI 1.15–2.5) in addition to a higher risk of caesarean birth in the early ARM group (25% versus 7.9%, RR 1.74; 95% CI 1.30–2.34) [9]. Lastly, a trial from India in which 150 women were randomised indicated shorter labours (7.35 h versus 11.66 h, *p* = 0.000) but a significant increase in caesarean rate (2.7% versus 10.7%, *p* = 0.049) when ARM was performed early [10].

A further two trials indicated that early and late amniotomy yielded equivalent outcomes between groups for rates of infection [11, 12]. The first of these trials was carried out in Canada and was unfortunately closed to recruitment at 143 participants after 3 years because of the low recruitment rate [11]. The primary outcome was rate of caesarean birth, which did not differ for nulliparous (18 vs 17%, *p* = 0.91) or parous (0 vs 3%, *p* = 1.0) people in the early and late amniotomy groups, respectively. This trial also indicated a non-significant trend towards fewer fevers in the early amniotomy group (3 vs 25%, *p* = 0.05). The second of these trials was performed in the United States and included 585 participants, all of them nulliparous [12]. The primary outcomes of the trial were time from induction to birth (19.0 vs 21.3 h, *p* = 0.04) and percentage of people who gave birth within 24 h (68 vs 56, *p* = 0.002) in the early and the late amniotomy groups, respectively. Chorioamnionitis rates were equivalent between groups (11.5 vs 8.5%, *p* = 0.22). However, in this trial, most people in the early ARM group had ARM at 3 cm cervical dilation, making the trial incomparable to the New Zealand context, where amniotomy is frequently performed as soon as feasible, which is at a cervical dilation that permits the insertion of an amniohook instrument (often 1–2 cm of dilation).

The findings of these trials are of significant import in the approach to IOL in New Zealand and internationally. Chorioamnionitis is a common occurrence in induced labour [8, 9]and is often a factor in the decision to perform an emergency caesarean birth and then in subsequent surgical infective complications. Chorioamnionitis entails maternal risks and requires treatment for both the mother and the infant during and after birth. Caesareans change the risks for the mother and infant but generally both have a higher risk of complications when a caesarean is performed after the commencement of labour (as occurs in the setting of labour induction). Chorioamnionitis is a risk factor for cerebral palsy and neonatal encephalopathy, even in term foetuses [13, 14].

Trial data regarding timing of amniotomy and infective and operative risk is mixed, showing either a benefit to delaying the procedure or no significant difference in outcomes between groups. There is some indication that induced labour may be slightly shorter with earlier amniotomy. None of the trials has been carried out in New Zealand, and both trials indicating equivalent outcomes in the early and late amniotomy groups were methodologically quite dissimilar to usual labour management in obstetric units in New Zealand. Indeed, the New Zealand national guideline on induction of labour identified timing of amniotomy as a research gap [15]. Irrespective, the procedure is performed on approximately 15,000 people who have IOL in New Zealand each year [15]. Therefore, ascertaining the ideal timing of ARM is important. To answer the question of whether early amniotomy causes an increased rate of chorioamnionitis in the setting of IOL, we are performing a randomised controlled trial. If our study indicates benefit to delaying amniotomy until active labour, this is a low-cost intervention (changing the timing of something that is done anyway) which could provide great benefit for women and babies.

### Objectives {7}

The objective is to assess the rate of chorioamnionitis in women undergoing early versus late ARM.

### Trial design {8}

The ARM trial is a randomised controlled trial being performed at a single institution in New Zealand, Te Toka Tumai Auckland. Participants undergoing oxytocin IOL are randomised to either ‘Early ARM’ or ‘Late ARM’ in a one-to-one ratio, stratified by parity (parity = 0 or ≥ 1). The primary hypothesis is that people undergoing ‘Late ARM’ will have a lower chance of developing chorioamnionitis than those people undergoing ‘Early ARM’.

## Methods: participants, interventions and outcomes

### Study setting {9}

This is a single-centre RCT, being carried out at Te Toka Tumai Auckland. The hospital is located in the central Auckland urban catchment and provides both assessment and birthing units as well as a level 3 NICU. Te Toka Tumai Auckland provides care to approximately 6000 people giving birth each year.

### Eligibility criteria {10}

Inclusion criteria:

- Pregnant people with a live singleton cephalic presentation
- Planning IOL at ≥ 37 weeks gestation
- Intact membranes
- Cardiotocography normal
- Require oxytocin for induction of labour

Exclusion criteria:

- Previous caesarean birth
- Major fetal congenital anomaly or known chromosomal abnormality
- Fetal growth restriction with absent or reversed end-diastolic flos noted on umbilical artery Doppler (fetal growth restriction with abnormal pulsatility index of the middle cerebral artery or umbilical artery or abnormal cerebroplacental ratio is permissable).

**Prior criterion was any participant in the OBLIGE study. This study is now completed [16]

### Who will take informed consent? {26a)

Patients identified as eligible are approached for inclusion for the trial when they present to the Women’s Assessment Unit at the Auckland City Hospital. Lead maternity carers (LMCs) who are either midwives or obstetricians, within the community, are aware of the trial and have frequently discussed it with the potential participant prior to their arrival to the unit. Once admitted for IOL, people are approached regarding the trial by either a research team representative or by an obstetric or midwifery team member. The trial procedures and purpose are reviewed with the patient, and a copy of the trial participant information sheet and consent form (PIS/CF) and pamphlet are provided for review. After an opportunity to discuss and ask questions, patients may choose to participate. Written informed consent is required for participation in the trial.

### Additional consent provisions for collection and use of participant data and biological specimens {26b}

N/A. Data will not be utilised in ancillary studies, and biological specimens are not collected.

## Interventions

### Explanation for the choice of comparators {6b}

The intervention groups in this trial are ‘Early’ and ‘Late’ timing of amniotomy. Standard care in our hospital is early amniotomy. The primary hypothesis is that people undergoing ‘Late ARM’ will have a lower chance of developing chorioamnionitis than those people undergoing ‘Early ARM’.

### Intervention description {11a}

Amniotomy is a common obstetric procedure during which a gloved hand is used to perform a vaginal examination. Once the cervix is located and the examiner’s fingers are placed inside the dilated portion, against the amniotic membrane, a plastic amnihook is advanced along the fingers. The instrument has a sharp ‘hook’ at the end. This end of the implement is utilised to cause a small tear in the amniotic membrane, after which time some of the amniotic fluid is usually felt to be expelled vaginally.

- ‘Early ARM’ group: ARM is performed either prior to or within 60 min of commencement of oxytocin infusion
- ‘Late ARM’ group: oxytocin infusion is commenced first, and ARM is performed at ≥ 6 cm cervical dilation or if the participant has been receiving oxytocin infusion for at least 12 h and has not yet reached 6 cm of cervical dilation

### Criteria for discontinuing or modifying allocated interventions {11b}

Participants may have modification to timing of amniotomy as the clinician caring for them sees fit. For example, a person allocated to having a ‘Late’ ARM may have this performed earlier than anticipated if a fetal scalp electrode needs to be placed for a clinical indication.

Participants can withdraw from study procedures at any time. This may include withdrawal for clinical procedures (for example requesting an ‘Early ARM’ though they are randomised to ‘Late ARM’) but with consent for continued use of data. Participants may also withdraw the use of their data from the trial. As a contingency for participant withdrawal from both clinical procedures and use of data, recruitment of 500 participants is planned (power calculation for the trial requires 488 participants).

### Strategies to improve adherence to interventions {11c}

Staff on the unit have had in-person education on the study groups. Additionally, written materials are available at all times on the delivery unit specifying the treatment per study arm.

### Relevant concomitant care permitted or prohibited during the trial {11d}

Clinical care during the trial is per hospital clinical guidelines.

### Provisions for post-trial care {30}

None outlined to participants. New Zealand has a public health care system. Treatment injuries (including injuries sustained during birth) are assessed and covered by the Accident Compensation Corporation (ACC) in New Zealand.

### Outcomes {12}

Primary outcome:

**Table:** 

Outcome measure	Timepoint	Method of ascertainment
Diagnosis of chorioamnionitis *Chorioamnionitis is defined as maternal temperature of greater than or equal to 38 °C OR 2 maternal temperatures of > 37.5 °C AND the choice to treat for chorioamnionitis with antibiotics	By birth of neonate	This outcome will be assessed via medical record review. Diagnosis and treatment of chorioamnionitis is recorded in the medical chart as well as the medication chart

Secondary outcomes:

**Table:** 

	**Outcome measure**	**Timepoint**	**Method of ascertainment**
1	Caesarean birth—overall incidence of caesarean birth post-induction	By birth of neonate	This outcome will be assessed via medical record review. An operative report will be recorded in the event that a caesarean is performed
2	Caesarean birth for fetal heart rate abnormality	By birth of neonate	This outcome will be assessed via medical record review. An operative report will be recorded in the event that a caesarean is performed
3	Caesarean birth for labour dystocia	By birth of neonate	This outcome will be assessed via medical record review. An operative report will be recorded in the event that a caesarean is performed
4	Fetal heart rate abnormalities	By birth of neonate	This outcome will be assessed via medical record review. An operative report with the operative indication(s) will be recorded in the event that a caesarean or instrumental vaginal birth is performed for fetal heart rate abnormalities. The midwife also performs a checklist after each birth which details the presence or absence of fetal heart rate abnormalities
5	Fetal heart rate abnormalities resulting in fetal scalp lactate sampling	By birth of neonate	This outcome will be assessed via medical record review. Fetal scalp lactate sampling and the results of the sampling are entered into the medical record
6	Fetal heart rate abnormalities resulting in instrumental vaginal delivery	By birth of neonate	This outcome will be assessed via medical record review. An operative report with the operative indication(s) will be recorded in the event that a caesarean or instrumental vaginal birth is performed for fetal heart rate abnormalities. The midwife also performs a checklist after each birth which details the presence or absence of fetal heart rate abnormalities
7	Maternal temperature greater than or equal to 38 °C	By birth of neonate	This outcome will be assessed via medical record review. The observation chart contains recordings of the maternal temperature readings
8	Maternal temperature of > 37.5 °C on single occasion during labour	By birth of neonate	This outcome will be assessed via medical record review. The observation chart contains recordings of the maternal temperature readings
9	Maternal temperature of > 37.5 °C on two occasions during labour	By birth of neonate	This outcome will be assessed via medical record review. The observation chart contains recordings of the maternal temperature readings
10	Postpartum endometritis	By diagnosis made within 7 days of birth	This outcome will be assessed via medical record review. The data will be available in the clinical record and treatment will be available in the medication chart
11	Infant born with 5-min Apgar scores < 7	By data imputed 5 min post-delivery	This outcome will be assessed via medical record review. The neonatal APGAR scores are recorded in the delivery summary
12	Infant born with abnormal cord blood lactate levels greater than or equal to 4.0	By data imputed from birth	This outcome will be assessed via medical record review. The neonatal cord blood gas readings are recorded in the delivery summary
13	Infants born with abnormal cord blood pH ≤ 7.10	By data imputed after delivery	This outcome will be assessed via medical record review. The neonatal cord blood gas readings are recorded in the delivery summary
14	Time to vaginal delivery	By birth of neonate	This outcome will be assessed via medical record review. The duration from start of oxytocin to delivery of the neonate will be calculated and presented as a median
15	Time from rupture of membranes to vaginal birth	By birth of the neonate	This outcome will be assessed via medical record review. The duration from start of rupture of membranes to the delivery of the neonate will be calculated and presented as a median
16	Neonatal intensive care unit (NICU) admission	By discharge from hospital of neonate	This outcome will be assessed via medical record review. NICU admission results in both a history and physical record and a discharge summary
17	Cost-effectiveness	By discharge of both mother and baby from hospital	This outcome will be assessed via medical record review. The cost of length of stay (in days) for both mother and baby will be assessed

### Participant timeline {13}

Person is identified as eligible.

↓

Discussion regarding study with clinician or member of the research team.

(after discussion with clinician).

Participant information sheet and consent form provided.

↓

Examination that assesses ARM feasible.

(either at presentation or after cervical preparation for induction of labour).

*People who go into labour from cervical preparation who no longer require oxytocin or with people who undergo SRM prior to randomisation are screen fails and are not randomised.

↓

Randomisation (online randomisation system [17]).

↓

IOL commences.

(*Early ARM group*: oxytocin infusion started and ARM within 60 min).

(*Late ARM group*: oxytocin started and ARM at 6 cm or more cervical dilation or at 12 h of oxytocin infusion).

↓

Participant completes post-induction survey regarding their birth experience.

↓

Data collection baseline demographics, labour and birth, maternal outcomes and neonatal outcomes to discharge from hospital.

### SPIRIT figure


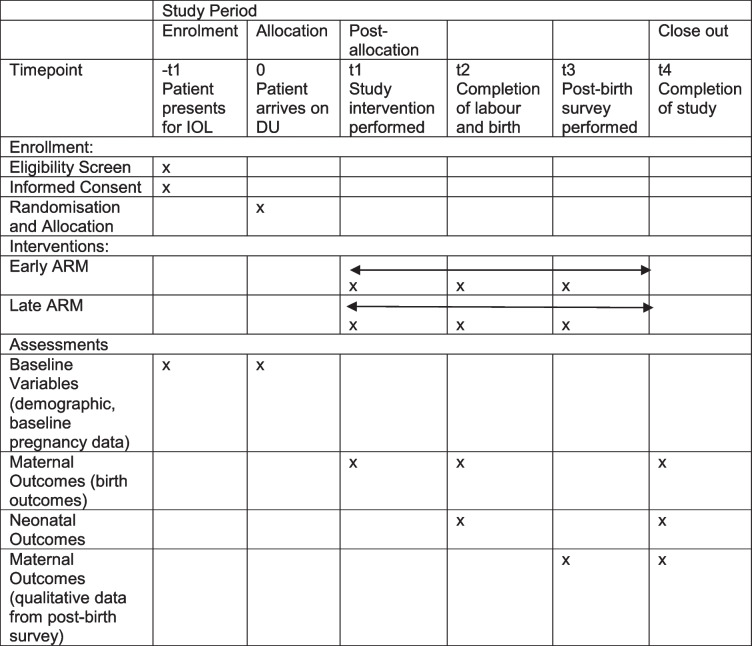


### Sample size {14}

A power calculation has been performed utilising the OpenEpi (https://openepi.com) program comparing the rates of chorioamnionitis between groups. The power calculation is based on the findings from the existing literature indicating a decrease in indicators of maternal infection when ‘Late ARM’ is performed [8, 9]. In order to show a decrease in chorioamnionitis from 9 to 3%, with power set at 80% and a 95% CI, 244 participants per arm are required, or 488 participants in total. The goal is to recruit 500 participants to this trial to account for participant withdrawals of data or loss to follow-up. The additional 12 participants are thought to be sufficient for the purposes of this trial as it would be highly unusual for a labouring patient to be ‘lost to follow-up’ for the primary outcome (which occurs in labour). We anticipate that the choice to withdraw data from analysis will be uncommon.

### Recruitment {15}

All eligible people and their care providers can access information about the study on the trial website ARM.auckland.ac.nz. Trial information sheets and trial pamphlets are available to potential participants when they present for induction as well as through the hospital clinics. There are research employees available to recruit for the trial through the assessment unit at the Auckland City Hospital. Participants have an in-person discussion regarding the trial with a member of staff or a member of the study team. At this time, they are provided with a participant information sheet and consent form to read. They are given time to review the materials and then may elect to participate. Written informed consent is required for participation.

## Assignment of interventions: allocation

### Sequence generation {16a}

Randomisation occurs via the Liggins institute Clinical Data Research Hub [17]. This service provides an electronic tool for participant screening and randomisation. The site contains a password-protected login, and the screening questions must indicate eligibility for the person to be randomised. Each patient identification number can only be randomised once within a 9-month period; hence, the randomisation cannot be performed more than once during the same pregnancy.

### Concealment mechanism {16b}

While there is allocation concealment until the point of the trial intervention being performed, blinding of participants and clinicians and data extractors is not feasible for this study. Data analysis will be blinded to study allocation.

### Implementation {16c}

The randomisation is computer generated and outsourced to the Liggins Institute Clinical Data Research Hub. The research midwives and investigators who enrol participants do not have access to the randomisation schedule. People can consent to the study at admission to the hospital for induction of labour. At Te Toka Tumai Auckland, IOL patients are initially seen in the Women’s Assessment Unit and cervical preparation undertaken in that location. When they are found to have a favourable cervical examination, they await transfer to delivery unit.

Once a participant arrives on delivery unit and is ready to commence induction with oxytocin, the midwife caring for the participant on delivery unit accesses the computerised randomisation site. The study intervention is then assigned electronically.

#### Screen failures

Participants in the study will frequently consent to inclusion either prior to or during cervical preparation for induction. Participants who have provided informed consent to be randomised who go on to labour with cervical preparation alone (thereby not requiring oxytocin infusion) or who undergo SRM (spontaneous rupture of membranes) prior to transfer to delivery unit are treated as ‘screen failures’ and are not randomised. While this may potentially result in a high rate of ‘screen failures’, the study was designed to have randomisation only once on the delivery unit thereby avoiding differential treatment of participants prior to oxytocin commencement based on study arm.

## Assignment of interventions: blinding

### Who will be blinded {17a}

It is not feasible to blind clinicians or participants to the study intervention. The statistician will be blinded to the study intervention during the analysis.

### Procedure for unblinding if needed {17b}

N/A. This study is not blinded.

### Data collection and management

#### Plans for assessment and collection of outcomes {18a}

Baseline data collection is from chart extraction.

Trial data collection is from chart extraction. Research employees performing chart extraction receive in-person training. Data collection is via electronic chart review. In relation to the primary outcome, vitals are reliably charted and the provision of antibiotics only occurs with a written record of the drug order. The secondary outcomes are all part of the standard data entry for all births (including for people not enrolled in the study) on our unit. Survey data is only completed either in person or via phone and is not available from the participant record.

Data is entered into a secure dataset.

#### Plans to promote participant retention and complete follow-up {18b}

There is 100% follow-up rate for participants for their immediate labour outcomes, as they remain on the unit for all study procedures. If any data is not able to be located within the chart, this data will be reported as missing for that individual outcome and not imputed. There is follow-up for a post-birth survey. This may be performed in person at a clinical encounter related to birth care (hospital or clinics) but is usually performed via phone. Attempts at contact are made three times via phone call for the post-birth survey.

#### Data management {19}

Data is entered into a secure dataset with identifiers removed. The dataset is accessible only to research staff. A separate list of participants is kept, only available to research staff.

#### Confidentiality {27}

Data is stored in a manner that has identifiers removed. The dataset of participants is kept on a secure server which is only accessible by study staff. All outputs from this research will be presented in an aggregated manner in a way that individual participants would not reasonably be able to be identified.

#### Plans for collection, laboratory evaluation and storage of biological specimens for genetic or molecular analysis in this trial/future use {33}

N/A. There are no biological specimens being collected as a part of this study.

## Statistical methods

### Statistical methods for primary and secondary outcomes {20a}

Descriptive data will be presented on the study groups.

Analyses will follow the principle of intention-to-treat. Missing data will not be imputed and will be presented as missing for these variables.

Additionally, a per-protocol analysis will also be undertaken, which will exclude from analysis any participant in the ‘Early’ ARM group who had ARM performed greater than 60 min after commencement of oxytocin infusion and any participant from the ‘Late’ ARM group who had ARM performed before 6 cm dilation or 12 h from oxytocin commencement due to trial procedure withdrawal. Participants who have protocol deviations for clinical indications (for example to place a fetal scalp electrode due to fetal heart rate abnormalities) will remain in the analysis.

Primary and secondary outcome analyses will be adjusted for the stratification variable using regression techniques, and outcomes presented as relative risks or mean differences with 95% confidence intervals.

A *p* value of 0.05 will be considered statistically significant. There are multiple secondary outcomes. These will be reported with *p* values without adjustment for multiplicity but recognised as exploratory.

### Economic analysis

The cost-effectiveness in this study will be determined via calculating length of stay in days for the pregnant person both antepartum and postpartum and the length of stay for the baby.

### Interim analyses {21b}

No interim analyses are planned. The data safety monitoring committee have access to limited outcome data and to severe adverse event data.

### Methods for additional analyses (e.g. subgroup analyses) {20b}

There are no additional analyses presently planned. We plan ongoing input from our Māori investigator and will re-discuss any additional analyses as we review the trial data. Any additional analyses will be added to the statistical analysis plan prior to data lock.

### Methods in analysis to handle protocol non-adherence and any statistical methods to handle missing data {20c}

Data for individual outcomes will not be imputed. Missing data percentages will be reported for each outcome.

### Plans to give access to the full protocol, participant-level data and statistical code {31c}

The full protocol is available on the Australian and New Zealand Clinical Trials Registry. Participants may opt-in to receiving a copy of study results when they consent to participate in this trial. The dataset will not be made publicly available.

## Oversight and monitoring

### Composition of the co-ordinating centre and trial steering committee {5d}

The trial steering committee is comprised of the investigators, who respond to any recommendations of the DSMC. In addition to these investigators, the trial group also relies on feedback from research midwives hired to perform study procedures.

### Composition of the data monitoring committee, its role and reporting structure (21a}

The DSMC is comprised of a neonatologist (chair) and an obstetrician. The DSMC receives reports each 25% of recruitment detailing recruitment, withdrawals, limited outcome data and severe adverse events. The DSMC makes recommendations to the trial steering committee regarding ongoing trial maintenance, any data requests and any additions to the factors reported. Statistical analyses for reporting to the DSMC are performed confidentially by a non-clinical co-investigator who is not involved in any aspect of trial recruitment, patient care, or data collection.

### Adverse event reporting and harms {22}

Adverse events (AEs) are collected for each participant. The primary outcome, chorioamnionitis, is considered an AE in labour, as are antepartum haemorrhage, postpartum haemorrhage (> 500 mL), umbilical cord prolapse, maternal birth injury, neonatal birth injury and neonatal infections requiring additional care (NICU admission or antibiotic treatment). The occurrence of instrumental vaginal birth and caesarean birth are also collected. These outcomes do not require contemporaneous reporting to the DSMC. These outcomes will be reported in the trial results. Unexpected events identified by the investigators or research staff are recorded with the adverse event data and reviewed by the PI for inclusion as adverse events.

Severe adverse events (SAEs) are reported to the PI and also to the DSMC. Each participant’s medical record is checked for the SAEs. Severe adverse events are specified as follows: maternal admission to intensive care unit or equivalent, maternal death, stillbirth, early neonatal death (defined as within 28 days), neonatal encephalopathy. The SAEs will be reported in the trial results.

### Frequency and plans for auditing trial conduct {23}

There are no planned audits.

### Plans for communicating important protocol amendments to relevant parties (e.g. trial participants, ethical committees) {25}

Major changes to the protocol need to be provided as an amendment to the ethics committee, reported to the clinical site, and the participant information materials need to be updated.

### Dissemination plans {31a}

Trial participants have the option of indicating their desire to receive trial results when they consent to participate in the trial.

The investigators intend to provide the results via local networks throughout New Zealand as well as internationally via conference presentations and publication in a peer-reviewed journal.

## Discussion

Recruitment for the trial commenced in June of 2021. During the first 18 months of the study, there were significant pandemic-related issues which impaired the recruitment rate. These included ‘silos’ of staff being created and discouragement of mixing across teams, hospital-wide shutdowns of research for weeks–months on three occasions and, lastly, inability of staff to perform recruitment as research was deemed a non-critical academic activity. Recruitment is now possible in the hospital, and staff have returned to study-related roles.

Internal barriers have been encountered, mostly related to the attitudes and beliefs of the clinical staff. The standard practice in New Zealand has for decades been the performance of amniotomy as soon as feasible, followed by oxytocin commencement. In spite of inconclusive data regarding duration of induction in the setting of amniotomy and the optimal timing of amniotomy, there is a lack of equipoise amongst the staff and potential recruiters. This has decreased buy-in from some lead maternity carers (LMCs) and their patients. Due to staff beliefs about timing of amniotomy during induction and labour duration, we have chosen hospital days as the element included in the cost-effectiveness analysis. One of the main factors causing care delays on our unit is lack of beds and suboptimal staffing of shifts. For this reason, length of stay is highly relevant locally.

A second barrier has been a commonly-held belief amongst both midwives and obstetricians that infusing oxytocin without performing an amniotomy increases the risk of amniotic fluid embolism (AFE). It is unclear what the basis for this belief is, but prior to trial start, the practice of early amniotomy was widely adhered to with the thought that it would prevent AFE. Now that educational sessions and individual discussions regarding the lack of evidence of risk with oxytocin infusion with intact membranes have been undertaken and the trial is underway with no findings to indicate a potential harm of delayed amniotomy, there has been increased momentum with recruitment.

Lastly, it has been noted that there have been several protocol violations in the ‘Late ARM’ group in the trial. This is for a variety of reasons, including misunderstanding of the trial protocol, participant request for amniotomy or staff obstetrician/LMC recommendation for amniotomy without a clear indication for this to be performed. This is not entirely unexpected, considering the strong belief amongst some care providers that amniotomy is important to induction of labour and the ability of participants to withdraw from the study at any time. Now that the trial is well underway and there has been ongoing unit and individual education about the trial, most people are treated per trial allocation. The investigators will perform a per-protocol analysis when recruitment is complete and the rate of protocol violations for both participant and caregiver-driven reasons is known.

## Trial status

Protocol version number: 2

Date recruitment began: 03 June 2021.

Approximate date recruitment will be complete: 30 May 2025.

## Data Availability

The investigators are willing to share any requested trial design data/materials with the journal.

## Abbreviations

ARM: Artificial rupture of membranes
IOL: Induction of labour
LMC: Lead maternity carer
NICU: Neonatal intensive care unit
PIS/CF: Participant information sheet and consent form
